# Clinical case presentation and a review of the literature of canine onchocercosis by *Onchocerca lupi* in the United States

**DOI:** 10.1186/s13071-015-0699-3

**Published:** 2015-02-08

**Authors:** Domenico Otranto, Alessio Giannelli, Nicole Scotty Trumble, Matt Chavkin, Gavin Kennard, Maria Stefania Latrofa, Dwight D Bowman, Filipe Dantas-Torres, Mark L Eberhard

**Affiliations:** Dipartimento di Medicina Veterinaria, Università degli Studi di Bari, Valenzano, Bari, Italy; BluePearl Veterinary Partners, Eden Prairie, Minnesota USA; VRCC Animal Eye Specialists, Englewood, Colorado USA; Albuquerque Practice, Albuquerque, New Mexico USA; Cornell University, Ithaca, New York USA; Departamento de Imunologia, Centro de Pesquisas Aggeu Magalhães (Fiocruz-PE), Recife, Pernambuco Brazil; Division of Parasitic Diseases and Malaria, Centres for Disease Control and Prevention, Atlanta, Georgia USA

**Keywords:** *Onchocerca lupi*, Canine onchocercosis, Zoonosis, United States, Clinical presentation

## Abstract

**Background:**

*Onchocerca lupi*, a filarioid of zoonotic concern, infects dogs and cats causing ocular lesions of different degrees, from minor to severe. However, infected animals do not always display overt clinical signs, rendering the diagnosis of the infection obscure to the majority of veterinarians. Canine onchocercosis has been reported in the Old World and the information on its occurrence in the United States, as well as its pathogenesis and clinical management is still meagre. This study reports on the largest case series of *O. lupi* infection from the United States and reviews previous cases of canine onchocercosis in this country.

**Methods:**

Information on the clinical history of a series of eight cases of *O. lupi* infection in dogs diagnosed in Minnesota, New Mexico, Colorado and Florida, from 2011 to 2014, was obtained from clinical records provided the veterinary practitioners. Nematodes were morphologically identified at species level and genetically analyzed.

**Results:**

All dogs displayed a similar clinical presentation, including subconjunctival and episcleral nodules, which were surgically removed. Each dog was subjected to post-operative therapy. Whitish filaria-like parasites were morphologically and molecularly identified as *O. lupi*.

**Conclusions:**

This study confirms that *O. lupi* is endemic in the United States, indicating that the distribution of the infection is probably wider than previously thought. With effect, further studies are urgently needed in order to improve the diagnosis and to assess the efficacy of therapeutic protocols, targeting the parasite itself and/or its endosymbionts.

## Background

Some filarioids (Spirurida, Onchocercidae) represent a major threat for human health, causing debilitating and socially stigmatizing life-threatening conditions, mainly in developing countries [1]. This is the case of *Wuchereria bancrofti* and *Onchocerca volvulus*, the agents of human lymphatic filariasis and river blindness, respectively, which are included in the WHO's priority list of the neglected tropical diseases [2,3]. Meanwhile, human infections by zoonotic filarioids are being more and more often reported worldwide [4], due to a plethora of environmental and anthropic factors allowing the spreading of arthropod vectors [5,6]. This has been recognized for the canine *Dirofilaria immitis* and *Dirofilaria repens*, whose mosquito vectors are prevalent in the same environments [5,7,8]. The role of dogs as reservoir hosts for zoonotic nematodes has spurred the interest of the scientific community towards the study of other species, which may parasitize domestic animals [4]. For example, *Onchocerca lupi* infects the ocular tissues of dogs and cats [9,10] causing from minor to severe ocular lesions [9,11]. In the initial stage, canine onchocercosis is associated with nonspecific ocular discomfort (i.e., excessive lacrimation, photophobia, conjunctivitis, exophthalmos and periorbital swelling) [9,12,13], while, in the chronic phase the typical nodules are detected on the external parts of the ocular apparatus (i.e., eyelids, nictitans, conjunctiva, and sclera) [9,14,15]. However, dogs do not always display overt clinical signs if *O. lupi* adults develop in the retrobulbar space of the eye [11,13]. Accordingly, the diagnosis of *O. lupi* relies on the detection of microfilariae in skin snips [16] and may also be investigated by the use of imaging tools (i.e., ultrasound scans, computed tomography) [14].

In the United States, cases of canine onchocercosis have been reported since the 90s in California, Utah and Arizona [17-19] but nematodes were not identified at species level. The tentative identification of the parasites in the cases above as *Onchocerca lienalis* has been questioned [12] based on the following circumstantial evidence: i) the site of the infection (i.e., filarioids were detected in the ocular region while *O. lienalis* localizes to the gastro-splenic ligament area of cattle); ii) the failure of experimental infection of dogs with this filarioid species [12]. In addition, the occurrence of gravid female nematodes in the patients above suggested that dogs were most likely the primary/proper host for those parasites, as is the case for *O. lupi*. The morphological and molecular identification of *O. lupi* in dogs and cats from the southwestern states (i.e., Colorado, California, Utah, Nevada) [10,20,21] raised the hypothesis that this parasite was implicated also in the cases above. Finally, the first zoonotic case of *O. lupi* in Arizona [21] has renewed the interest about this parasite, which has also been increasingly found in humans in the Old World [22-25].

The information on the pathogenesis of canine onchocercosis is still minimal and little data is available on the clinical management of *O. lupi*. Therefore, this article documents the clinical presentation and the treatment of a series of recently reported cases of canine onchocercosis from the Unites States (i.e., Minnesota, New Mexico, Colorado and Florida) [26], along with a review of canine onchocercosis in this country.

## Methods

### Clinical history and case outcome

Information on the clinical history of a series of eight cases of *O. lupi* infection in dogs diagnosed in the Minnesota, New Mexico, Colorado and Florida, from 2011 to 2014, was obtained from clinical records provided by the veterinary practitioners in charge for each case. Information on treatment and case outcome was also provided. All medical procedures were performed after obtaining the owner consent.

Cases on canine onchocercosis diagnosed in the United States, published in the literature were also reviewed. In particular, data on the sex, age, geographical origin, travel history, localization of the nodules and parasite identification for each individual case was analyzed from articles published in the international literature, from 1991 to 2014 [12,17-20,26].

### Morphological and molecular identification

Nematodes collected were morphologically identified at species level based on [27] and [22]. In addition, specimens extracted from dogs 2 and 3 were molecularly processed. Following the genomic DNA extraction by DNeasy Blood & Tissue Kit (Qiagen, GmbH, Hilden, Germany), partial *cytochrome oxidase subunit* 1 (*cox*1) and 12S rDNA genes were amplified and sequenced as described elsewhere [22].

## Results

### Clinical history and case outcome

#### Case 1

An 8-year-old female dog, rescued in Hollywood (Florida) on June 2014, was presented to a local ophthalmologist due to the onset of conjunctival granulomas, which were surgically removed and histologically examined. The animal was treated for heartworm with ivermectin (400 μg/kg orally every day, for 30 days) and fenbendazole (100 mg/kg orally once daily). A few months later, conjunctival lesions reoccurred OU and the animal underwent a second surgery during which nematodes were extracted. Samples were sent to Cornell University to be morphologically and molecularly identified. A gravid female worm was morphologically identified as *Onchocerca* sp. and microfilariae sent to the Centers for Disease Control and Prevention (CDC; Atlanta, GA, USA).

#### Case 2

**Figure 1:**
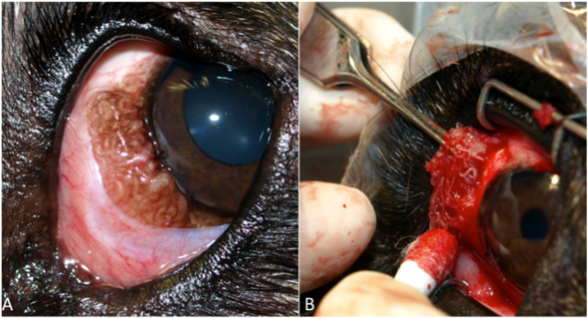
**Ophthalmic examination of dog 1 with subconjunctival masses containing convoluted nematodes (A); surgical removal of the nematodes (B).**

A 7-year-old castrated mixed breed male dog from Oronoco (Minnesota) presented at the BluePearl Veterinary Partners hospital in Eden Prairie (Minnesota) with a 1-year history of red eyes that worsened into proliferative ocular lesions on the medial aspects of the globes OU. The owners reported that their pet was adopted one year before from the municipality of Durango (Colorado) and that it suffered mild scleritis. At the ophthalmic examination, both eyes appeared similar with two pink subconjunctival masses. The first (5 mm in diameter) was located immediately posterior to the nasal limbus, and the second, less dense, was located approximately 3 mm posterior to the temporal limbus (Figure 1A). Since the administration of ivermectin (450 μg/kg orally once daily), fenbendazole (100 mg/kg orally once daily), and corticosteroids did not resolve the ocular lesions, surgical excision of the masses was undertaken. Whitish filaria-like parasites were removed from the sites, the majority of which were embedded within the bulbar conjunctiva, sclera and extraocular muscles (Figure 1B). Post-operative therapy included cefpodoxime (5 mg/kg orally once daily for 10 days), carprofen (2.2 mg/kg orally twice daily for 5 days then once daily for 5 days), and tramadol (2 mg/kg orally one to three times daily), as well as ivermectin (600 μg/kg orally) and fenbendazole (100 mg/kg orally) both once daily for 8 weeks. The eyes were reportedly normal with no recurrence of lesions reported during six weeks of post-operative follow-up and four months of post-operative contact with the primary veterinarian and owners.

#### Case 3

**Figure 2:**
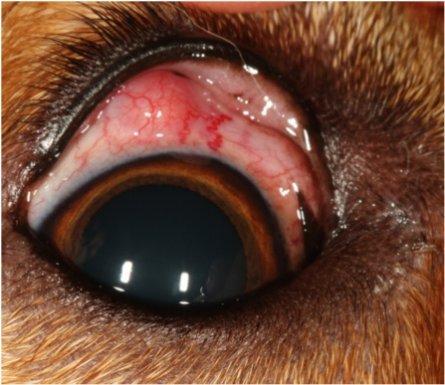
**Ophthalmic examination of dog 2.** Red swollen left eye.

A 6-year-old neutered female Labrador retriever mix dog was presented on March 2014 at the Veterinary Specialty & Emergency Hospital in Englewood (Colorado). The animal displayed a red swollen left eye (Figure 2), with squinting and tearing from both eyes. The patient was originally from Farmington (New Mexico) and suffered from red tearing eyes in the past. The animal was heartworm antigen negative. During the ophthalmic examination, episcleral swelling and episcleral vascular congestion was reported in the temporal quadrant OS, as well as scleral indentation of the temporal fundus. Whitish nematodes were detected during the incisional biopsy and stored in 70% ethanol, to be morphologically and molecularly identified. An oral treatment with doxycycline (10 mg/kg orally once daily for three months) and ivermectin (400 μg/kg orally once daily for two months then sporadically for a total of five months) was undertaken. The animal was re-examined 6 months after and a complete resolution in the ocular discomfort was recorded.

#### Case 4

A 2-year-old female mix breed was examined on June 2014. The dog was originally from Farmington (New Mexico), where she was adopted about six weeks before. The animal was apparently healthy, apart for a slight squinting and disconformity in the size of the eyes. The animal was heartworm antigen negative. At the ophthalmic examination, the eyes presented numerous inflammatory conjunctival follicles, mild epiphora, mild diffuse conjunctival hyperemia and soft episcleral swellings in the temporal quadrant, larger in the right eye. During the incisional biopsy, some nematodes were detected and extracted. Oral treatment with ivermectin (400 μg/kg orally once a month for six months) and doxycycline (100 mg/kg orally once daily for three months) was performed. The animal was followed-up three months later and did not show any relapse.

#### Case 5

**Figure 3:**
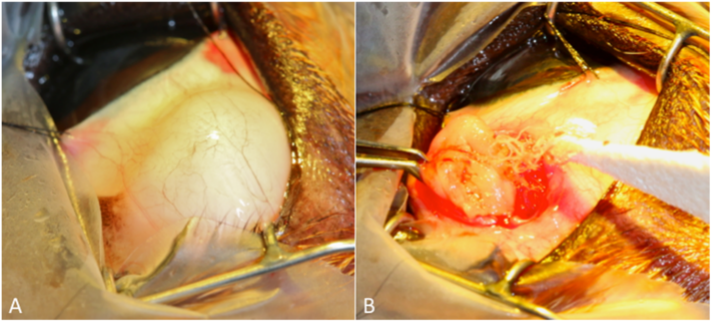
**Ophthalmic examination of dog 3.** Evident episcleral nodule **(A)** and nematodes extracted during the incisional biopsy **(B)**.

A 3-year-old neutered male Labrador retriever mix dog was presented on October 2012 with a complaint of recent conjunctivitis in both eyes. The animal was rescued from an animal shelter in Farmington (New Mexico), approximately one year before. At the presentation, no blepharospasm or significant conjunctival hyperemia were observed but large subconjunctival granulomas (Figure 3A), containing multiple intertwined white thread-like nematodes, were noted in the dorsal bulbar region in both eyes. The animal underwent preoperative treatment with moxydectin (0.22 mg/kg subcutaneously), prednisone (0.5 mg/kg orally twice daily), doxycycline (5 mg/kg orally twice daily) and neopolydex in both eyes. Subconjunctival exploratory surgery was performed one week later (Figure 3B) and nematodes were extracted. Following the surgery, melarsomine (2.5 mg/kg intramuscular) was administered in the lumbar epaxial muscle and the patient was treated with prednisone (0.5 mg/kg orally twice daily for one week following exploratory, tapered to once daily), doxycycline (5 mg/kg orally twice daily) and neopolydex in both eyes, three times daily. The animal was heartworm antigen negative. Post-operative recheck examinations were performed once each month for four months; at each examination during this four-month period, an injection of moxydectin (0.22 mg/kg subcutaneously) was given. At the end of this four-month period, prophylactic oral antihelmintic therapy was initiated at 400 μg/kg orally every day for 30 days. Recheck examinations were again performed at six months and at one year. No further instances of episcleral masses were noted.

#### Case 6

A 3-year-old neutered male Yorkshire terrier dog from Farmington (New Mexico) was presented on May 2014 due to moderate chemosis and to an episcleral flocculent mass in the lateral portion in the right eye. The animal was heartworm antigen negative. A subconjunctival exploratory was performed and multiple thread-like nematodes were removed from the episcleral region in the right eye. The animal underwent the same therapeutic protocol and recheck examinations of case 4, being completely healthy at each follow-up.

#### Case 7

**Figure 4:**
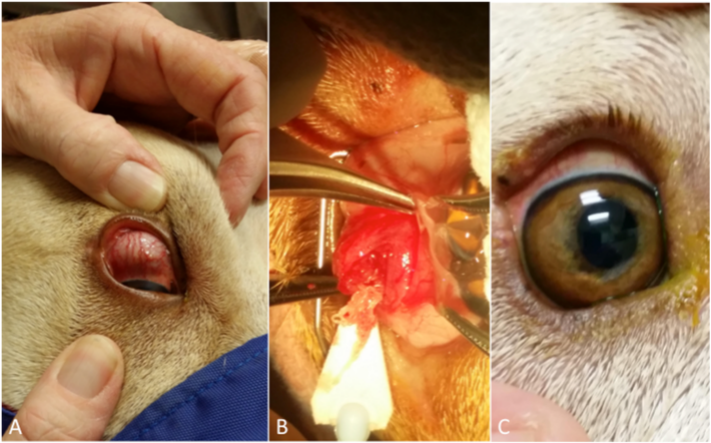
**Ophthalmic examination of dog 4.** Episcleral nodule **(A)** and nematodes extracted during the incisional biopsy **(B)**. Two weeks post surgery follow-up with complete resolution of the ocular condition **(C)**.

A 9-year-old neutered female red Australian cattle dog from Jerez (New Mexico) was presented on June 2013 with a history of a chronic waxing/waning episcleral mass in the left eye, which was firm on palpation (Figure 4A). The animal was heartworm antigen negative and was treated with doxycycline (5 mg/kg orally twice daily), antibiotics and corticosteroids. One week after the presentation, the owner reported that rapid and significant response to the medical therapy was achieved, with complete resolution of the episcleral mass. Therefore, the anti-inflammatory and antibiotic treatment was suspended six weeks after the presentation. Six months later, the episcleral mass reoccurred and the animal was taken to surgery (Figure 4B). At time of surgery, the animal underwent the same therapeutic protocol and recheck examinations of case 4 and 5 and, one month after, complete resolution of the episcleral mass was noted in the left eye. However, a generalized episcleritis was recorded in the right eye, associated with a moderate exophthalmos. Therefore, a second subconjunctival exploratory was performed in the right eye and multiple nematodes were extracted. The animal was treated as in the cases above. A last follow up three months after surgery revealed complete resolution of the ocular alterations.

#### Case 8

A 5-year-old neutered male German shepherd dog from Farmington (New Mexico) was presented on April 2011 due to chronic conjunctivitis in the left eye. Ophthalmic findings revealed a large flocculent episcleral mass in the left eye, occupying the entirety of the ventral episcleral mass and extending distally past the equatorial portion of the globe. The animal was diagnosed with keratoconjunctivitis sicca (KCS) but was heartworm antigen negative. A subconjunctival exploratory surgery was then performed and several thread-like nematodes were removed from the left eye. The animal was treated as in the cases above, along with tacrolimus (0.03% in both eyes, twice daily) to address the KCS and was monthly rechecked for a period of four months. One year following the last monthly recheck examination, the dog displayed recurrence of conjunctivitis in the left eye, exhibiting moderate chemosis of the ventral bulbar conjunctiva. A second subconjunctival exploratory surgery was performed but no nematodes were detected. Monthly recheck examinations were performed for a period of three months. At each of these rechecks moxydectin was administered (0.22 mg/kg subcutaneously). Additional postoperative therapy was doxycycline, doxycycline (5 mg/kg orally twice daily for three months) and neopolydex in both eyes, three times daily. Following monthly recheck examinations and the final moxydectin injection, prophylactic oral anthihelmintic therapy with ivermectin was initiated at 400 μg/kg orally every day for 30 days and recommended for life. The dog remained disease free.

### Morphological and molecular identification

Specimens presented external round transverse ridges and two transverse striae per each outer ridge interval, with a typical body diameter/ridge distance interval of 7–10:1 [27]. The nematode morphology and the sequencing of barcoding genes of parasites obtained from cases 2 and 3 were concordant in identifying the specimens recovered from all cases herein reported as *O. lupi*. In addition, a 100% nucleotide sequence homology was recorded between p*cox*1 [26] and 12S rDNA sequences herein obtained (AN: KP283474 and KP283475) and those of specimens collected from dogs and cats from different states within the United States (*cox*1: JX080028- JX080031, JF758473-JF758475, KC763785-KC763786; 12S rDNA: KC763783).

## Discussion

**Figure 5:**
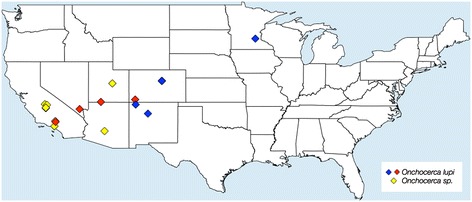
**Distribution of canine onchocercosis in the USA.** Blue diamonds indicate new cases of *Onchocerca lupi* infection.

Data reported provide new information on the clinical presentation of *O. lupi* infection in dogs. Along with data in the literature (Table 1; Figure 5), results confirm that *O. lupi* is widespread in the United States. Indeed, all previous descriptions of canine onchocercosis caused by unidentified *Onchocerca* species [12,17-19], may be attributed to *O. lupi* due to the site of the infection (i.e., ocular tissues), which is peculiar to this nematode, and to a retrospective analysis of the geographical origin of the infections. For example, *O. lupi* has been diagnosed in Palmdale, California [20], which is situated only 12 km away from the municipality of Lancaster where a case of *Onchocerca* sp. was previously documented [19].

**Table 1:** **Canine onchocercosis in the USA: review of cases described in the literature and present report, along with data on the geographical origin, location of lesions and diagnosis**

**Sex (Age in years)**	**Geographic origin (Travel)**	**Localization of the nodule (eye)**	**Diagnosis**	**Reference in the text**
Female (15)	California	Sclera (OD, then OS)	*Onchocerca* sp.	[17]
Female (10)	Los Angeles, California	Sclera	*Onchocerca* sp.	[18]
Male (9)	Utah	Conjunctiva, Sclera (OS, then OD)	*Onchocerca* sp.	[18]
Male (adult)	Phoenix, Arizona	Free worm in the cornea	*Onchocerca lienalis*	[19]
Female (1)	Lancaster, California	Retrobulbar space (OD)	*Onchocerca lienalis*	[19]
Male (6)	California	Sclera	*Onchocerca* sp.	[12]
Male (10)	California (Washington, Idaho, Nevada)	Sclera (OS)	*Onchocerca* sp.	[12]
Male (adult)	Palmdale, California	Conjunctiva	*Onchocerca lupi*	[20]
Male (adult)	Las Vegas, Nevada	-	*Onchocerca lupi*	[20]
Male (adult)	Mancos, Colorado	Conjunctiva	*Onchocerca lupi*	[20]
Male (adult)	Kanab, Utah	-	*Onchocerca lupi*	[20]
Male (7)	Oronoco, Minnesota (Durango, Colorado)	Conjunctiva (OU)	*Onchocerca lupi*	[26]
Female (6)	Englewood, Colorado (New Mexico)	Sclera (OS)	*Onchocerca lupi*	[26]
Female (2)	Englewood, Colorado (Farmington, New Mexico)	Sclera (OU)	*Onchocerca lupi*	[26]
Male (3)	Farmington, New Mexico	Conjunctiva (OU)	*Onchocerca lupi*	[26]
Male (3)	Farmington, New Mexico	Sclera (OD)	*Onchocerca lupi*	[26]
Female (9)	Jemez, New Mexico	Sclera (OS, then OD)	*Onchocerca lupi*	[26]
Male (5)	Farmington, New Mexico	Sclera (OS, relapsing)	*Onchocerca lupi*	[26]

Consequently, the epidemiology of *O. lupi* in the United States deserves to be thoroughly assessed since it is likely more widely distributed than currently believed. Indeed, all the reports above were characterized by nodular lesions, but, in the only large epidemiological study performed in Portugal and Greece, where clinical cases were reported, up to 8.4% of the examined animals scored positive for *O. lupi* microfilariae [11]. Certainly, a serological tool for the diagnosis of *O. lupi* [28] would overcome the difficulties related with the detection of microfilariae in the skin snip biopsy, which is an invasive procedure and of limited usefulness during the prepatent period of the infection. Today, the skin-snip for the detection of skin-dwelling microfilariae is the most sensitive procedure for the diagnosis in asymptomatic dogs. This tool should be used for screening dogs relocated from endemic areas, such as New Mexico, to other regions.

Cases herein reported were characterized by a similar clinical presentation, which included subconjunctival and episcleral nodules. In addition, all dogs examined were adults (from three to nine years of age), which may be due to the fact that the incubation period of *O. lupi* infection is usually long [9], as already described for *O. volvulus* (i.e., up to 18 months) [2]. The negative results obtained with the heartworm antigen tests suggest that commercial kits used for screening *D. immitis* infection are not able to detect cross-reactions with *O. lupi*. Accordingly, since some of the dogs herein examined underwent chemoprophylactic treatment for the canine heartworm disease with ivermectin, this therapy is most likely ineffective against *O. lupi*. Indeed, to date the only treatment with reputed efficacy for ocular onchocercosis is represented by the surgical removal of the parasitic nodule followed by post-operative therapeutic administration of melarsomine (2.5 mg/kg intramuscularly, daily for two days), ivermectin (50 μg/kg subcutaneously, one month after the initial treatment), topical antibiotics and systemic prednisolone [12,14]. The above pharmaceutical treatment has been successfully applied to control the relapse of nodules up to more than one year of follow-up. However, although *Wolbachia* endosymbionts were detected in *O. lupi* [10,15,29] any chemoprophylactic combination of tetracyclines with macrocyclic lactones has never been tested. Indeed, the use of doxycycline targeting the *Wolbachia* endosymbionts of filariae was effective in damaging and even killing *D. immitis* adult worms [30] and succeeded in eliminating adults of *D. immitis*, eventually reducing the risk of thromboembolism [31,32]. Similarly, a combination of doxycycline hyclate (10 mg/kg twice daily for 30 days) and ivermectin (6 μg/kg every 15 days for six months) was effective for treating microfilariaemia in dogs affected by *D. repens,* a cause of subcutaneous dirofilariosis [33].

## Conclusion

Data herein reported indicate that *O. lupi* may be a risk for human health. Along with the human case reported from Arizona [21], other cases of human onchocercosis have been diagnosed in Arizona, Colorado, Minnesota and Pennsylvania [21,34]. Therefore, further studies are urgently needed in order to improve the diagnosis of *O. lupi* infection infection, to better define its pathogenesis, and to report the efficacies of therapeutic protocols, based on controlled clinical trials. These studies should target the parasite itself and/or its endosymbionts.

## References

[1] Orihel TC, Eberhard ML (1998). Zoonotic filariasis. Clin Microbiol Rev.

[2] CDC Global health (2013). Available at http://www.cdc.gov/parasites/lymphaticfilariasis/. Accessed 14 June 2013.

[3] Evans DS, Alphonsus K, Umaru J, Eigege A, Miri E, Mafuyai H (2014). Status of Onchocerciasis transmission after more than a decade of mass drug administration for onchocerciasis and lymphatic filariasis elimination in central Nigeria: challenges in coordinating the stop MDA decision. PLoS Negl Trop Dis.

[4] Otranto D, Dantas-Torres F, Brianti E, Traversa D, Petrić D, Genchi C (2013). Vector-borne helminths of dogs and humans in Europe. Parasit Vectors.

[5] Genchi C, Mortarino M, Rinaldi L, Cringoli G, Traldi G, Genchi M (2011). Changing climate and changing vector-borne disease distribution: the example of *Dirofilaria* in Europe. Vet Parasitol.

[6] Otranto D, Eberhard ML (2011). Zoonotic helminths affecting the human eye. Parasit Vectors.

[7] McCall JW, Genchi C, Kramer LH, Guerrero J, Venco L (2008). Heartworm disease in animals and humans. Adv Parasitol.

[8] Pampiglione S, Rivasi F, Gustinelli A (2009). Dirofilarial human cases in the Old World, attributed to *Dirofilaria immitis*: a critical analysis. Histopathology.

[9] Sréter T, Széll Z (2008). Onchocercosis: a newly recognized disease in dogs. Vet Parasitol.

[10] Labelle AL, Daniels JB, Dix M, Labelle P (2011). *Onchocerca lupi* causing ocular disease in two cats. Vet Ophthalmol.

[11] Otranto D, Dantas-Torres F, Giannelli A, Latrofa MS, Papadopoulos E, Cardoso L (2013). Zoonotic *Onchocerca lupi* infection in dogs, Greece and Portugal, 2011–2012. Emerg Infect Dis.

[12] Zarfoss MK, Dubielzig RR, Eberhard ML, Schmidt KS (2005). Canine ocular onchocerciasis in the United States: two new cases and a review of the literature. Vet Ophthalmol.

[13] Franchini D, Giannelli A, Di Paola G, Cortes H, Cardoso L, Lia RP (2013). Image diagnosis of zoonotic onchocercosis by *Onchocerca lupi*. Vet Parasitol.

[14] Komnenou A, Eberhard ML, Kaldrymidou E, Tsalie E, Dessiris A (2002). Subconjunctival filariasis due to *Onchocerca* sp. in dogs: report of 23 cases in Greece. Vet Ophthalmol.

[15] Komnenou A, Egyed Z, Sréter T, Eberhard ML (2003). Canine onchocercosis in Greece: report of further 20 cases and molecular characterization of the parasite and its *Wolbachia* endosymbiont. Vet Parasitol.

[16] Otranto D, Dantas-Torres F, Giannelli A, Abramo F, Ignjatović Ćupina A, Petrić D (2013). Cutaneous distribution and circadian rhythm of *Onchocerca lupi* microfilariae in dogs. PLoS Negl Trop Dis.

[17] Orihel TC, Ash LR, Holshuh HJ, Santenelli S (1991). Onchocerciasis in a California dog. Am J Trop Med Hyg.

[18] Gardiner CH, Dick EJ, Meininger AC, Lozano-Alarcón F, Jackson P (1993). Onchocerciasis in two dogs. J Am Vet Med Assoc.

[19] Eberhard ML, Ortega Y, Dial S, Schiller CA, Sears AW, Greiner E (2000). Ocular *Onchocerca* infections in two dogs in western United States. Vet Parasitol.

[20] Labelle AL, Maddox CW, Daniels JB, Lanka S, Eggett TE, Dubielzig RR (2013). Canine ocular onchocercosis in the United States is associated with *Onchocerca lupi*. Vet Parasitol.

[21] Eberhard ML, Ostovar GA, Chundu K, Hobohm D, Feiz-Erfan I, Mathison BA (2013). Zoonotic *Onchocerca lupi* infection in a 22-month-old child in Arizona: first report in the United States and a review of the literature. Am J Trop Med Hyg.

[22] Otranto D, Sakru N, Testini G, Gürlü VP, Yakar K, Lia RP (2011). Case report: First evidence of human zoonotic infection by *Onchocerca lupi* (Spirurida, Onchocercidae). Am J Trop Med Hyg.

[23] Otranto D, Dantas-Torres F, Cebeci Z, Yeniad B, Buyukbabani N, Boral OB (2012). Human ocular filariasis: further evidence on the zoonotic role of *Onchocerca lupi*. Parasit Vectors.

[24] Ilhan HD, Yaman A, Morishima Y, Sugiyama H, Muto M, Yamasaki H (2013). *Onchocerca lupi* infection in Turkey: a unique case of a rare human parasite. Acta Parasitol.

[25] Mowlavi G, Farzbod F, Kheirkhah A, Mobedi I, Bowman DD, Naddaf SR (2013). Human ocular onchocerciasis caused by *Onchocerca lupi* (Spirurida, Onchocercidae) in Iran. J Helminthol.

[26] Otranto D, Giannelli A, Latrofa MS, Dantas-Torres F, Scotty Trumble N, Chavkin M, et al. Canine Infections with *Onchocerca lupi* Nematodes, United States, 2011–2014. Emerg Infect Dis. 2015 (accepted for publication). 10.3201/eid2105.141812 PMC4412234 25897859

[27] Mutafchiev Y, Dantas-Torres F, Giannelli A, Abramo F, Papadopoulos E, Cardoso L (2013). Redescription of *Onchocerca lupi* (Spirurida: Onchocercidae) with histopathological observations. Parasit Vectors.

[28] Giannelli A, Cantacessi C, Graves P, Becker L, Campbell BE, Dantas-Torres F (2014). A preliminary investigation of serological tools for the detection of *Onchocerca lupi* infection in dogs. Parasitol Res.

[29] Egyed Z, Sréter T, Széll Z, Nyiro G, Márialigeti K, Varga I (2002). Molecular phylogenetic analysis of *Onchocerca lupi* and its *Wolbachia* endosymbiont. Vet Parasitol.

[30] Kramer L, Grandi G, Leoni M, Passeri B, McCall J, Genchi C (2008). *Wolbachia* and its influence on the pathology and immunology of *Dirofilaria immitis* infection. Vet Parasitol.

[31] Bazzocchi C, Mortarino M, Grandi G, Kramer LH, Genchi C, Bandi C (2008). Combined ivermectin and doxycycline treatment has microfilaricidal and adulticidal activity against *Dirofilaria immitis* in experimentally infected dogs. Int J Parasitol.

[32] Grandi G, Quintavalla C, Mavropoulou A, Genchi M, Gnudi G, Bertoni G (2010). A combination of doxycycline and ivermectin is adulticidal in dogs with naturally acquired heartworm disease (*Dirofilaria immitis*). Vet Parasitol.

[33] Giannelli A, Ramos RA, Traversa D, Brianti E, Annoscia G, Bastelli F (2013). Treatment of *Dirofilaria repens* microfilariaemia with a combination of doxycycline hyclate and ivermectin. Vet Parasitol.

[34] Biswas A, Yassin MH (2013). An unexpected cause of eye irritation: a case of zoonotic ocular onchocerciasis. Case Rep Infect Dis.

